# Peritoneal Cavity Is Dominated by IFNγ-Secreting CXCR3^+^ Th1 Cells

**DOI:** 10.1371/journal.pone.0018032

**Published:** 2011-07-18

**Authors:** Beata M. Zygmunt, Lothar Groebe, Carlos A. Guzman

**Affiliations:** 1 Department of Vaccinology and Applied Microbiology, Helmholtz Centre for Infection Research, Braunschweig, Germany; 2 Department of Experimental Immunology, Helmholtz Centre for Infection Research, Braunschweig, Germany; Cleveland Clinic, United States of America

## Abstract

The chemokine receptor CXCR3, which was shown to take part in many inflammatory processes, is considered as a Th1 specific marker. Here, we show in a mouse model that CXCR3 expressing CD4^+^ cells preferentially migrate to the peritoneal cavity under steady-state conditions. The peritoneal cavity milieu leads to an up-regulated expression of CXCR3. However, blocking of known ligands of this chemokine receptor did not alter the preferential migration. The peritoneal cavity environment also results in an increased percentage of memory cells producing cytokines. Up-regulation of IFNγ production occurs mostly in CXCR3^+^ cells considered as Th1, whereas the up-regulation of IL-4 affects mostly in CXCR3^−^ cells which are considered as Th2. We conclude that the peritoneal cavity does not change the Th-lineage of the cells, but that domination of this anatomic niche by Th1 cells rather results from preferential migration to this compartment.

## Introduction

The T-helper (Th) bias of the lymphocytes is known to be crucial for the outcome of immune responses against infection, as it has been shown in the *Leishmania major* system, where the cytokine profile is the most important determinant of successful clearance of the pathogen [1]. There are many factors influencing the shape of the Th immune response and the cytokine profile of stimulated CD4^+^ T cells [2], [3]. In this context, INFγ, IL-4 and IL-17 are considered as a signature cytokines for Th1, Th2 and Th17 cells, respectively. However, recent data suggest that there is a considerable plasticity of Th cells in respect to their capacity to produce cytokines [4], [5], [6].

T cells properties vary considerably among different anatomical compartments. Interestingly, recent data suggest that the tissue microenvironment influences the gene expression profile of immune cells, independently of the variables associated with the specific priming in lymphoid organs. It has been shown that the lung environment in the absence of cognate Ag alters the expression level of activation markers like CD69 and CD127 [7]. There is also a report about a special CD8^+^ memory cell pool from the gut epithelium, resembling neither central nor effector memory cells [8]. The unique phenotype of these cells arises *in situ* within the gut, and is modified upon changes in location. The tissue microenvironment can also influence the TLR expression pattern by CD11c^+^ cells [9]. The transcriptional profiles [10], expression levels of surface molecules and homing phenotype of B1 cells is also changed in the peritoneal cavity (PerC) [11].

The Th subsets differ not only by cytokine production patterns, but also in the expression of homing molecules, such as the chemokine receptors. IFNγ producing Th1 cells, which are mainly involved in immune responses against intracellular pathogens, are characterized by the expression of CXCR3 and CCR5. Th2 cells, which secrete IL-4, IL-5 and IL-13 and take part in immune responses against extracellular bacteria and parasites, express on their surface CCR4 [12]. The IL-17-producing Th17 lineage, which was reported to be involved in the defense against certain bacterial pathogens and fungi [13], [14], is characterized by the expression of CCR6 [14]. However, the association of a chemokine receptor with a Th lineage is not strict. Th1 and Th2 cells can also express chemokine receptors that are characteristic for the other subsets, although certain combinations of chemokine receptors are markedly enriched in these cell populations and define specific Th subsets [15]. *In vitro* functional studies showed that both Th1 and Th 2 cells can migrate towards CXCL9 (CXCR3 ligand) and CCL17 (CCR4 ligand), but the migration of Th1 towards CXCL9 and Th2 towards CCL17 is more efficient [16]. Requirement for IFNγ and STAT1, the signaling molecule involved in Th1 differentiation, for induction of CXCR3, and IL-12 and STAT4 for CCR5 expression indicate the association of these chemokine receptors with Th1 immune response [17], [18].

CXCR3 belongs to the group of inflammatory cytokine receptors. Beside Th1 cells, it is expressed on subsets of B cells secreting IgG antibodies [12], [19]. Involvement of CXCR3 in CD4^+^ lymphocytes migration was shown in numerous pathological conditions. CXCR3 and its ligands participate in immune response against *Bordetella bronchiseptica* [20]. It has also a non redundant role in T cell migration to dermal inflammation sites [21]. The blockage of CXCR3 receptor ligands reduces leukocyte recruitment to the lung in the model of idiopathic pneumonia syndrome [22], and diminishes recruitment of Th1 cells to the inflamed peritoneum [23].

In this study we are showing that there are different factors which may influence the Th-composition of a particular territory. Our results demonstrate that Th1 cells preferentially migrate to PerC and that this territory is dominated by Th1 cells. Moreover, the PerC environment increases the expression levels of different cytokines, including IFNγ.

## Materials and Methods

### Mice

BALB/c and C57BL/6 mice were purchased from Harlan (Borchen, Germany) and were used at the age 8 to 16 weeks. All animal experiments have been performed in accordance with institutional guidelines and have been approved by the local government of lower Saxony (animal permission 33.11.42502-04-104/07).

### Antibodies

The following antibodies have been used: anti-CD4-FITC, anti-CD4-PE, anti-CD4-PerCP, anti-CD4-APC-Cy7 and anti-IFNγ-PE from BD Bioscience; anti-IL-4-PE-Cy7, anti-IL-17-APC and anti-IFNγ-APC from eBioscience; anti-CXCR3-PE and anti-CXCR3-APC from R&D Systems.

### Cell isolation

In all the experiments mice were euthanized by CO_2_ inhalation. Lymphoid organs were dissected and single-cell suspensions were obtained by mincing organs through a 100 µm nylon mesh. For isolation of lymphocytes from lungs, mice were first perfused with 20 ml of PBS by heart puncture and tissues were then mechanically disrupted. Erythrocytes were lyzed where necessary by using the ACK buffer. For isolation of peritoneal cells, the PerC was flushed with 10 ml of PBS.

### Flow cytometry and cell sorting

Cells were stained with the antibodies and data acquisition was performed using a FACSCanto (BD Biosciences). Data were analyzed using FACSDiva (BD Bioscience) or FlowJo (Tree Star, Ashland, OR). Cells were sorted on a MOFlo (Dako Cytomation) or FACSAria (BD Bioscience). In some experiments, before sorting CD4^+^ T cells were isolated using the CD4^+^ T Cell Isolation Kit (Miltenyi Biotec), according to the manufacturer's protocol.

### Adoptive transfer experiments

For competitive adoptive transfer experiments CD4^+^ CXCR3^+^ and CD4^+^ CXCR3^−^ or CD4^+^ CXCR3^+^ CD62L^low^ CD44^high^ and CD4^+^ CXCR3^−^ CD62L^low^ CD44^high^ cells were obtained by sorting cells isolated from spleens (Sp) or PerC of naïve BALB/c or C57BL/6 mice. Cells were stained with 1 µM CFSE in PBS for 5 min or with 10 µM CMTMR in PBS for 15 min (Molecular Probes). The reactions were stopped by adding equal volumes of pre-warmed FCS and further incubated for 5 min. Cells were washed in medium and re-suspended in PBS. Different populations of cells were mixed in a 1∶1 ratio and injected in the tail vein of naïve recipient animals. The composition of the cell suspensions was controlled by FACS and any inequality in cells proportions were complied in calculations. To exclude the possibility that observed differences in cell migration were due to unspecific effects resulting from the fluorescent staining procedure, different combinations of CFSE- and CMTMR-labeled cells were used. The animals were sacrificed after 18 h and the numbers of transferred cells were determined in different lymphoid and non-lymphoid organs by flow cytometry.

For adoptive transfer experiments splenocytes or sorted CD4^+^ CXCR3^+^ CD62L^low^ CD44^high^, CD4^+^ CXCR3^−^ CD62L^low^ CD44^high^ and CD4^+^ CXCR3^−^ CD62L^high^ CD44^low^ cells were stained with 1 µM CFSE in PBS for 5 min. The reactions were stopped by adding equal volumes of pre-warmed FCS and further incubated for 5 min. Cells were then washed in medium and re-suspended in PBS. Cell suspensions were injected i.v. or intraperitonealy (i.p.) to recipient animals. After 24 or 36 h cells were re-isolated from the Sp or PerC of the animals. Re-stained cells were analyzed for the expression level of CXCR3 by flow cytometry.

### Cytokine neutralization

For cytokine neutralization studies mice received 40 µg of anti-CXCL9, 100 µg of anti-CXCL10 and 100 µg of anti-CXCL11 (RnD Systems) by i.v. route 4 h before adoptive transfer. The control animals received appropriate amounts of isotype control antibodies.

### Cytokine assessment

Sorted cells were incubated in RPMI medium supplemented with 10% FCS, 100 units/ml of penicillin-streptomycin, 2 mM L-glutamine (Gibco), 1 µg/ml ionomycin and 0.01 µg/ml PMA (Sigma) for 24 h. Supernatants were collected and stored in −70. Cytokines were evaluated by ELISA (eBioscience), according to manufacturer's protocol.

### Intracellular cytokine staining

Cells were incubated in RPMI medium supplemented with 10% FCS 100 units/ml of penicillin-streptomycin, 2 mM L-glutamine (Gibco) in the presence of 1 µg/ml ionomycin and 0.01 µg/ml PMA (Sigma) for 4 h. For the last 2 h of culture Brefeldin A (Sigma) was added to the final concentration of 5 µg/ml. After washing, cells were stained with antibodies against surface markers and fixed for 20 min in 2% PFA. For intracellular cytokine staining cells were incubated on ice for 30 min in permeabilization buffer (0.5% saponin and 0.5% BSA in PBS). After washing, cells were stained with antibodies in permeabilization buffer for further 30 min. After 2 additional washing steps in permeabilization buffer and one in PBS, cells were analyzed by flow cytometry.

### Statistical analysis

Statistical analysis was performed using unpaired t test. Values of p<0.05 were considered significant.

## Results

### The percentage of CXCR3^+^ CD4^+^ cells is higher in PerC than in other organs

**Figure 1:**
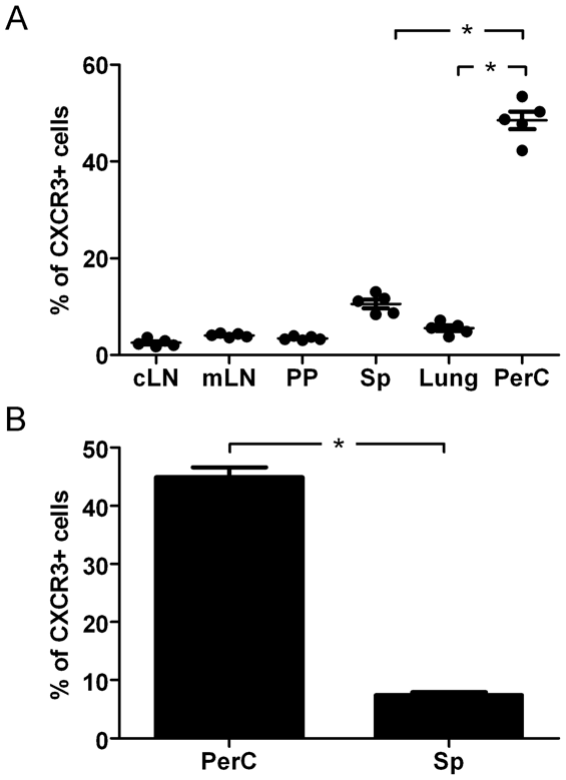
The percentage of CXCR3^+^ CD4^+^ T cells is higher in PerC than in other organs. The percentage of CXCR3^+^ CD4^+^ T cells in different organs was determined by flow cytometry. (A) Cells isolated from naïve Balb/c animals were analyzed (n = 5). Each point represents an individual animal. (B) Cells isolated from 10 to 20 naïve Balb/c animals were pooled. Results from 8 independent experiments are presented. Error bars indicate SEM. cLN, cervical lymph node; mLN, mesenteric lymph node; PP, Peyer's patches; Sp, spleen; PerC, peritoneal cavity. (*) Statistically significant at P<0.05.

We first investigated the proportion of CXCR3^+^ CD4^+^ cells in different organs. Cells isolated from BALB/c animals were stained to analyze the expression of CXCR3 by FACS. The results showed that the proportion of CXCR3^+^ cells among CD4^+^ cells is significantly higher in PerC than in any other organ tested (Figure 1A). The data obtained from over 100 animals showed that the average percentage in PerC is 45% in comparison to 7% in the Sp (Figure 1B). These results are not strain specific, since similar values were observed in C57BL/6 mice (data not shown).

### CXCR3^+^ CD4^+^ T cells migrate preferentially to PerC

**Figure 2:**
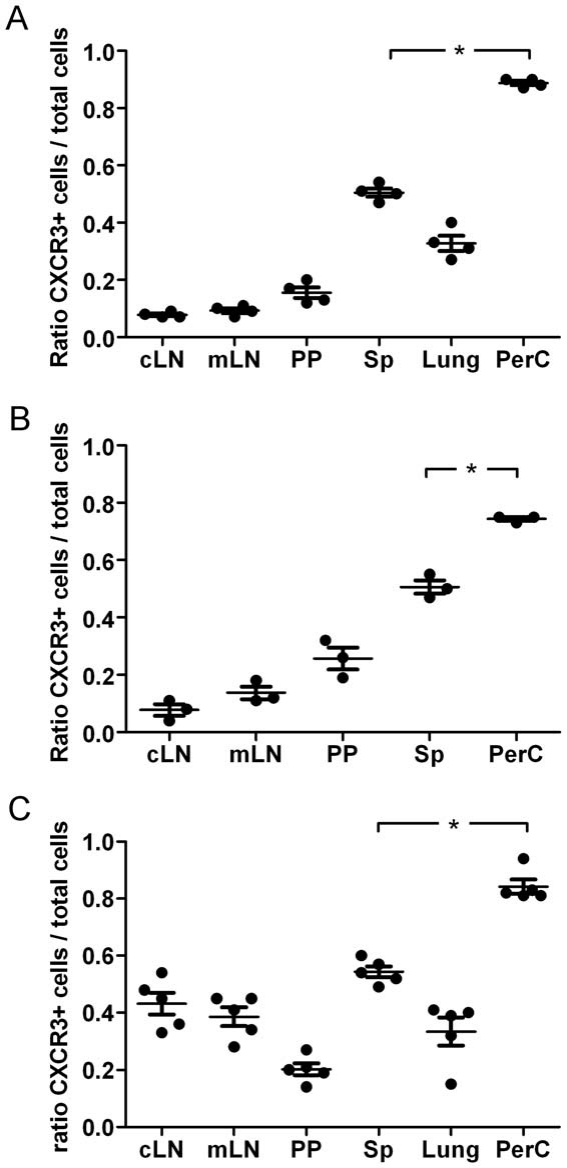
CXCR3^+^ CD4^+^ T cells preferentially migrate to the PerC. CXCR3^+^ and CXCR3^−^ CD4^+^ T isolated from donor animals were differentially labeled with CFSE or CMTMR, and mixtures containing equal numbers of cells were injected intravenously into naïve recipients. Eighteen hours after transfer, the ratio of transferred CXCR3^+^ cells to total transferred (CXCR3^+^+CXCR3^−^) cells was determined in different organs by flow cytometry. (A) CD4^+^ CXCR3^+^ and CD4^+^CXCR3^−^ T cells isolated from Sp of donor BALB/c mice. (B) CD4^+^ CXCR3^+^ and CD4^+^ CXCR3^−^ T cells isolated from PerC of donor BALB/c mice. (C) CD4^+^ CXCR3^+^ CD62L^low^ CD44^high^ and CD4^+^ CXCR3^−^ CD62L^low^ CD44^high^ T cells isolated from Sp of C57BL/6 donor mice. Dots represent the ratio for the individual animals analyzed. Similar results were obtained in three independent experiments. Horizontal lines indicate the mean for the group, error bars correspond to the SEM. (*) Statistical significance at P<0.001. cLN cervical lymph node; mLN, mesenteric lymph node; PP Peyer's patches; Sp, spleen; PerC, peritoneal cavity.

This observation led us to ask if CXCR3^+^ CD4^+^ cells preferentially migrate to PerC. To answer this question, we sorted CXCR3^+^ and CXCR3^−^ CD4^+^ cells from Sp of BALB/c mice and differentially stained them with CFSE and CMTMR, respectively. Cells were then mixed in a 1∶1 ratio and injected i.v. to recipient animals. After 18 h cells were re-isolated from different organs and the proportion of transferred CXCR3^+^ to total transferred cells was analyzed by flow cytometry (Figure 2A). This experiment showed that there is strong preference for CXCR3^+^ cells to migrate to PerC. In contrast, both cell populations showed a similar homing to Sp. In the other examined organs, the percentage of CXCR3^+^ cells was below 50% of total transferred cells. We repeated this experiment using cells sorted from PerC and obtained similar results (Figure 2B). The results for lungs are not shown, due to the low number of cells migrating to this organ. The CXCR3^+^ CD4^+^ cell population consists almost exclusively from memory cells defined as a CD44^high^ CD62L^low^, whereas the CXCR3^−^ CD4^+^ cell population contains both memory and naïve cells [15]. Thus, we decided to assess if the observed phenomenon resulted from differences in migration for these two cell populations. To this end, we sorted memory CXCR3^+^ and CXCR3^−^ CD4^+^ memory cells (defined as CD44^high^ CD62L^low^ cells) from Sp of naïve C57BL/6 animals and performed an adoptive transfer, as previously described (Figure 2C). C57BL/6 mice were chosen for this experiment, since up-regulation of CD44 in BALB/c mice renders this marker not useful for memory cells identification in this strain. The obtained results confirmed that CXCR3^+^ cells preferentially migrate to the PerC. However, we have not observed a strong migration of CXCR3^−^ cells to lymph nodes like in the previous experiments. Since migration of naïve cells to lymph nodes but not to Sp is dependent on CD62L, we assume that the observed differences are due to the absence of the CD62L^high^ subpopulation in the CXCR3^−^ cells.

### The PerC milieu increases the expression of CXCR3 on CD4^+^ T cells

**Figure 3:**
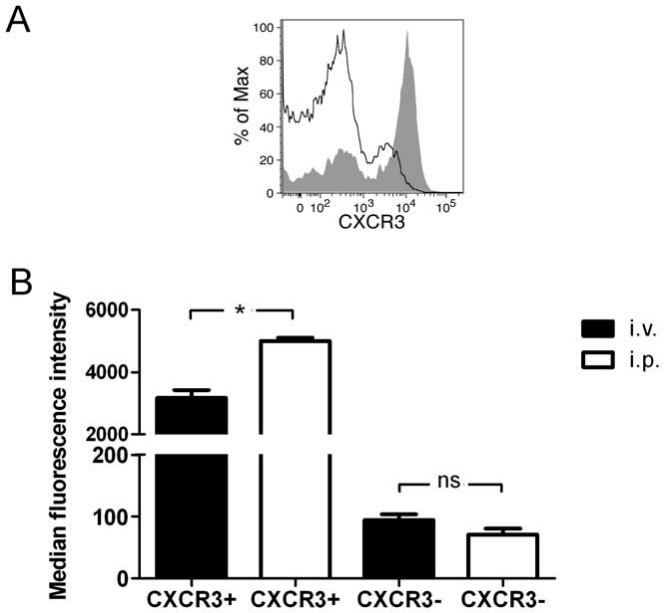
The PerC environment results in increased expression of CXCR3 on CD4^+^ T cells. (A) The expression level of CXCR3 on CD4^+^ cells from spleen (black line) or PerC (grey area) of BALB/c mice was determined by flow cytometry. (B) CXCR3^+^ and CXCR3^−^ CD4^+^ T cells were sorted from Sp of donor BALB/c mice and stained with CFSE. Cells were then adoptively transferred to naïve recipient animals by i.v or i.p. route. After 24 h, cells were re-isolated from spleens or peritoneal cavities and re-stained with an anti-CXCR3 antibody. The expression level of CXCR3 on CFSE^+^ cells was then analyzed by flow cytometry. The differences were statistically significant at P<0.001 (*), n.s. – not significant.

During our experiments we observed that the expression level of CXCR3 is higher on cells isolated from PerC (Figure 3A) than on Sp cells. There are reports showing that the PerC environment can lead to changes in gene expression profiles [10], expression of surface molecules and homing phenotype of B1 cells [11]. Thus, we decided to evaluate if this is also the case for CD4^+^ cells, thereby resulting the differences in the expression levels of CXCR3 to an imprinting occurring in the PerC. To this end, we sorted CXCR3^+^ and CXCR3^−^ CD4^+^ cells from BALB/c mice and stained them with CFSE. Then, the cells were separately injected to recipient animals by i.v. or i.p. route. After 24 h cells were re-isolated from Sp or PerC of i.v. or i.p. injected animals, and after re-staining they were analyzed by flow cytometry. The obtained results showed that CXCR3 is up-regulated on CXCR3^+^ cells which are exposed to the PerC environment (Figure 3B). However, there was no statistically significant difference in CXCR3 expression between CXCR3^−^ cells isolated from Sp and PerC (Figure 3C). We conclude that the PerC environment increases the expression level of CXCR3 on CD4^+^ cells positive for this molecule, but it does not affect the frequency of CXCR3^+^ CD4^+^ cells.

### The preferential migration of CD4^+^ cells to PerC is not dependent on CXCR3

**Figure 4:**
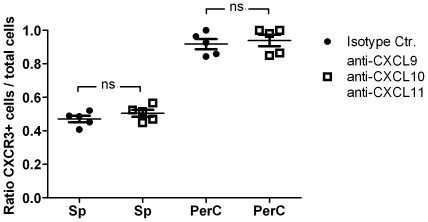
Neutralization of CXCR3 ligands does not block preferential migration of CXCR3^+^ CD4^+^ T cells to the PerC. CXCR3^+^ and CXCR3^−^ CD4^+^ T cells isolated from donor animals were differentially labeled with CFSE or CMTMR, and mixtures containing equal numbers of cells were injected intravenously into naïve recipients pretreated with a cocktail of anti-CXCL9, anti-CXCL10 and anti-CXCL11 antibody or an appropriate isotype control. Eighteen hours after transfer, the ratio of transferred CXCR3^+^ cells to total transferred cells was determined in different organs by flow cytometry. Dots represent the ratio for individual animals. Horizontal lines indicate the mean for the group, and error bars correspond to the SEM. n.s. – not significant. Sp, spleen; PerC, peritoneal cavity.

To assess if the preferential migration of CD4^+^ CXCR3^+^ cells to the PerC is dependent on the expression of this chemokine receptor, we performed a competitive adoptive transfer experiment in which known ligands of CXCR3 were blocked with CXCL9, CXCL10 and CXCL11 specific antibodies. No significant differences were observed in the migration pattern of transferred cells (Figure 4). This suggests that CXCR3 is not directly responsible for the observed migration.

### Peritoneal CD4^+^ T cells preferentially re-enter the PerC

**Figure 5:**
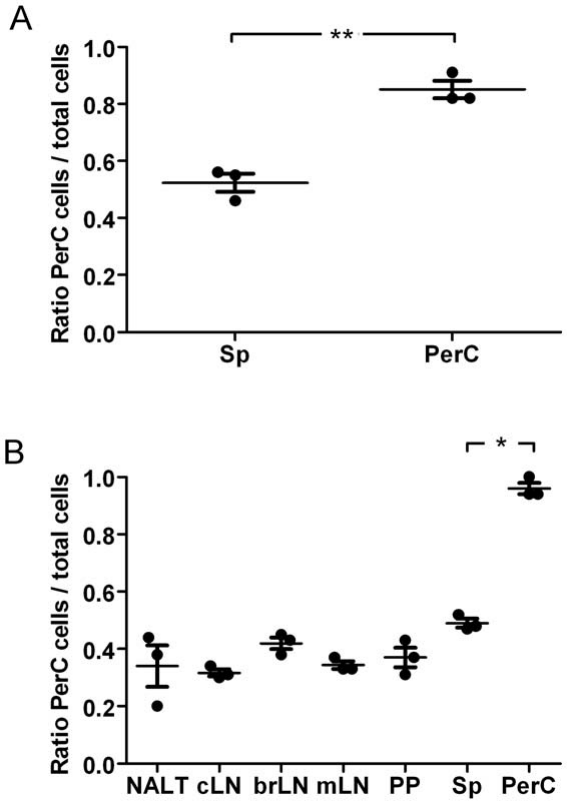
Peritoneal CD4^+^ T cells preferentially re-enter the peritoneal cavity. (A) CXCR3^+^ or (B) CXCR3^−^ CD4^+^ T cells isolated from spleen or peritoneal cavity were differentially labeled with CFSE or CMTMR, and mixtures containing equal numbers of cells were injected intravenously into naïve recipients. 18 hours after transfer, the ratio of transferred peritoneal cells to total transferred cells (peritoneal cells+spleen cells) was determined in different organs by flow cytometry. Similar results were obtained in three independent experiments. Dots represent the ratio for individual animals. Horizontal lines indicate the mean for the group, and error bars correspond to the SEM. The differences were statistically significant at P<0.0001 (*) and P<0.002 (**). NALT, nasal associated lymphoid tissues; cLN, cervical lymph node; brLN, bronchial lymph node; mLN, mesenteric lymph node; PP, Peyer's patches; Sp, spleen; PerC, peritoneal cavity.

The data reported here showed that the PerC environment leads to up-regulation of CXCR3 on CXCR3^+^ cells. In addition, previous studies demonstrated that this is also the case for the expression of other surface molecules, which are involved in B1 cell homing [11]. Thus, we assessed if the exposition to the PerC environment changes the migratory properties of CD4^+^ cells. To do this, we sorted CXCR3^+^ CD4^+^ cells from Sp and PerC of donor mice. The cells were differentially stained with CFSE or CMTMR, mixed in equal proportions and injected i.v. to recipient animals. After 18 h, cells were re-isolated and the proportion of peritoneal to total transferred cells in PerC and Sp of acceptor animals was determined by flow cytometry (Figure 5A). This experiment showed that there is a preferential migration of cells exposed to PerC environment to this compartment. On the other hand, our studies suggest that CXCR3 is not responsible for the preferential homing to PerC. Thus, we performed this experiment using also CXCR3^−^ cells isolated from either PerC or Sp. Again, we observed a preferential migration of cells isolated from PerC to its origin compartment (Figure 5B). This suggests that the imprinting of cells is taking place regardless of CXCR3 expression.

### Th cells are differentially distributed

**Figure 6:**
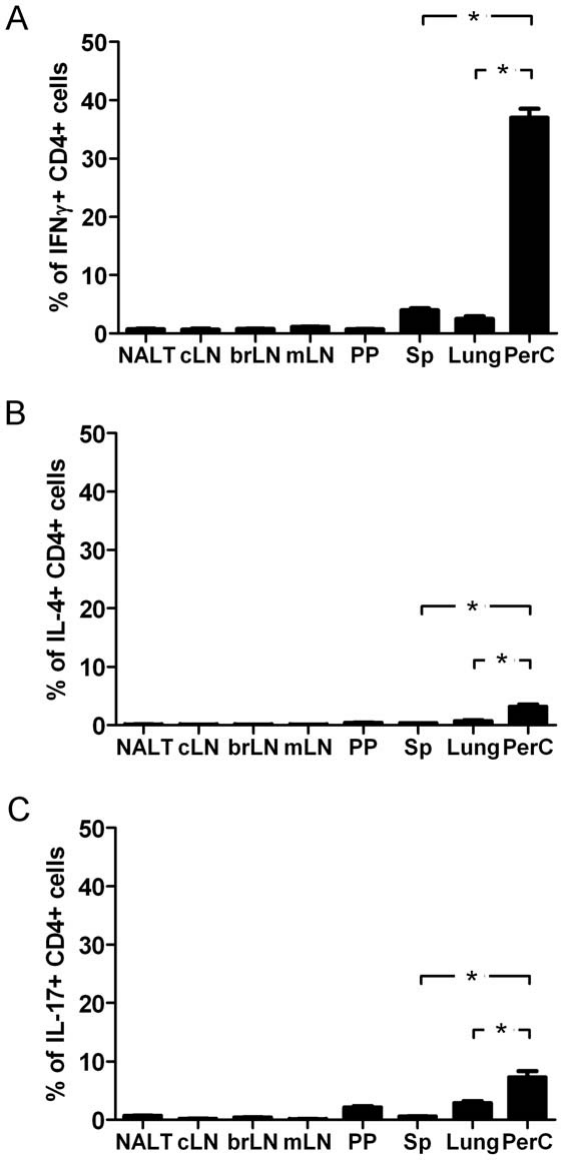
Th cell subsets are differentially distributed in different anatomic niches. The percentage of different Th cell subsets in CD4^+^ cell population were determined by FACS analysis in different organs (n = 5). (A) Th1 cells (IFNγ secreting cells), (B) Th2 cells (IL-4 secreting cells) and (C) Th17 cells (IL-17 secreting cells). The differences were statistically significant at P<0.001 (*). Similar results were obtained in 2 independent experiments. NALT, nasal associated lymphoid tissues; cLN, cervical lymph node; brLN, bronchial lymph nodes; mLN, mesenteric lymph node; PP, Peyer's patches; Sp, spleen; PerC, peritoneal cavity.

Our results showed that in comparison to other anatomic niches, the PerC is dominated by CXCR3^+^ CD4^+^ cells. IFNγ is mostly produced by CXCR3^+^ CD4^+^ cells, thereby suggesting that this compartment is predominantly populated by Th1 cells. To validate this hypothesis, cells isolated from different organs were stained for cytokines and analyzed by flow cytometry. Our hypothesis was fully confirmed by the obtained experimental data (Figure 6A). The proportion of IFNγ secreting cells present in the PerC is many times higher than in any other organ tested. Of remark, the proportion of Th1 cells in the PerC is also much higher in comparison to lungs, another peripheral territory tested. We also assessed the expression of other cytokines by peritoneal CD4^+^ cells (Figure 6B and C). It was observed that the percentage of cells expressing IL-4 or IL-17 is also higher in the PerC than in other organs, however, the differences are not as prominent as for IFNγ.

### The PerC environment increases the expression of IFNγ by CD4^+^ T cells

**Figure 7:**
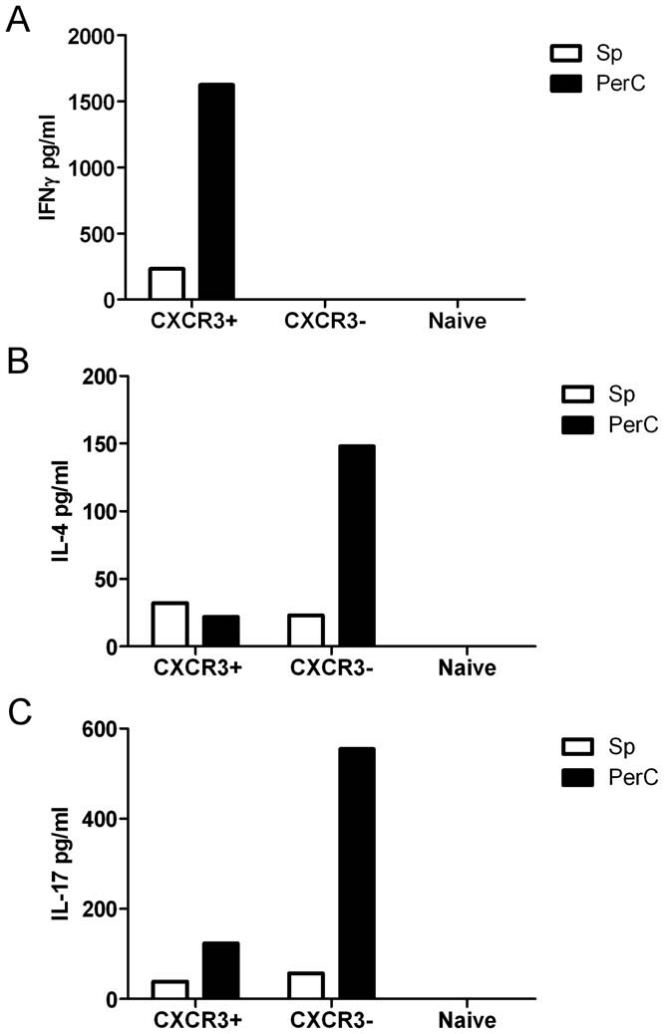
Peritoneal memory CD4^+^ T cells produce more cytokines than Sp derived cells. CXCR3^+^ CD62L^low^ CD44^high^, CXCR3^−^ CD62L^low^ CD44^high^ and CXCR3^−^ CD62L^high^ CD44^low^ CD4^+^ T cells were sorted from Sp or PerC of C57BL/6 naïve (specific pathogen free) mice. Then, cells were re-stimulated with ionomycin and PMA and the expression of (A) IFNγ, (B) IL-4 and (C) IL-17 was measured by ELISA. Similar results were obtained in 2 independent experiments.

Taking into account that exposition to the peritoneal milieu results in changes in the expression levels of certain molecules, a comparative evaluation of the cytokines produced by peritoneal and Sp cells was performed. We sorted CXCR3^+^ CD62L^low^ CD44^high^, CXCR3^−^ CD62L^low^ CD44^high^ and CXCR3^−^ CD62L^high^ CD44^low^ CD4^+^ cells. The levels of cytokine production were then measured by ELISA in culture supernatants of stimulated cells (Figure 7). The obtained results showed that, as expected, IFNγ is produced by CXCR3^+^ cells, whereas IL-4 and IL-17 are mostly expressed by CXCR3- cells. When the expression levels of the two populations were compared, by and large cells isolated from the PerC showed the highest expression levels.

**Figure 8:**
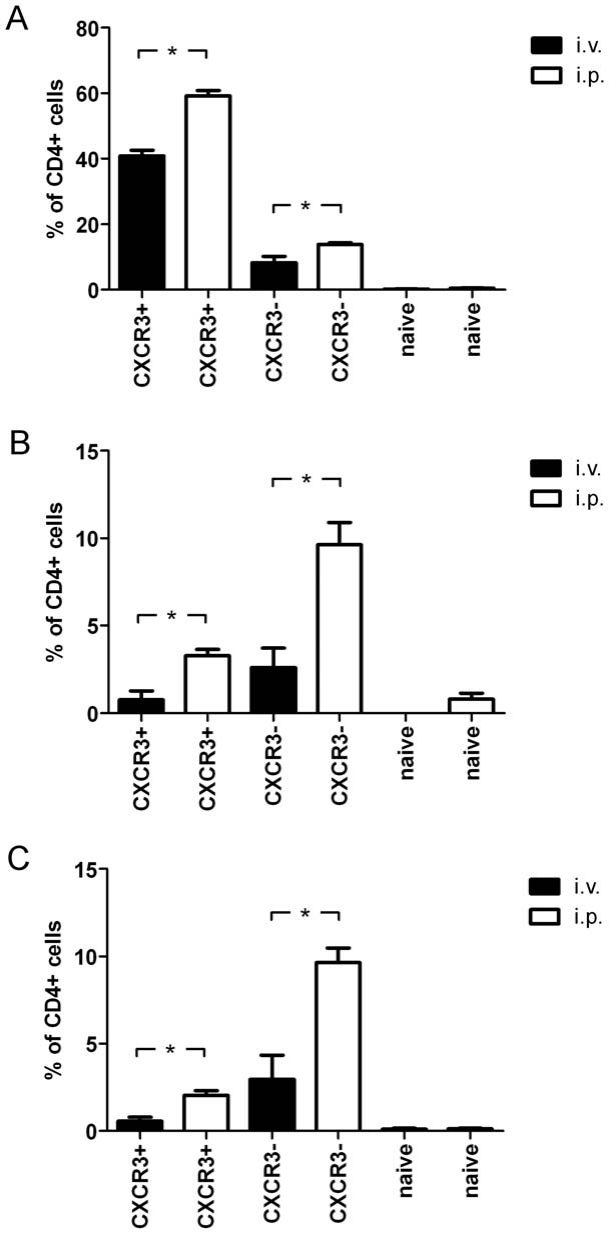
The environment of the PerC results in increased expression of IFNγ by CD4^+^ T cells. CXCR3^+^ CD62L^low^ CD44^high^, CXCR3^−^ CD62L^low^ CD44^high^ and CXCR3^−^ CD62L^high^ CD44^low^ CD4^+^ T cells were sorted from Sp of naïve C57BL/6 mice and stained with CFSE. Then, cells were transferred to naïve recipient animals by i.v. or i.p. rout and re-isolated after 24 h. Cells were re-stimulated with ionomycin, PMA and brefeldinA, stained for (A) IFNγ, (B) IL-4 and (C) IL-17 production and further analyzed by flow cytometry. The percentage of cells producing the different cytokines was subsequently determined. Similar results were obtained in 2 independent experiments. The differences were statistically significant at P<0.01 (*).

To assess if the observed differences are related to the influence of the peritoneal milieu, we sorted CXCR3^+^ CD62L^low^ CD44^high^, CXCR3^−^ CD62L^low^ CD44^high^ and CXCR3^−^ CD62L^high^ CD44^low^ CD4^+^ T cells from Sp of naïve C57BL/6 mice. Then, cells were stained with CFSE and subsequently transferred to naïve recipients animals by i.v. or i.p. route. After 24 h cells were re-isolated, re-stimulated with ionomycin, PMA and brefeldinA, stained for IFNγ, IL-4 and IL-17 production and analyzed by flow cytometry. The obtained results showed that the percentage of transferred cells producing a particular cytokine is always higher in case of cells which were re-isolated from PerC than from Sp (Figure 8 A to C).

## Discussion

Our initial observation that PerC is dominated by CXCR3^+^ CD4^+^ cells leads us to investigate the migratory behavior of these cells. We showed that CXCR3^+^ cells under steady state conditions migrate more efficiently to this territory than CXCR3^−^ CD4^+^ cells. The observed differences are not due to the memory phenotype of CXCR3^+^ lymphocytes, since these cells exhibit preferential migration to this compartment also when compared to CXCR3^−^ memory cells. Since CXCR3 is known to be a proinflammatory chemokine receptor and its involvement in the migration of CD4^+^ lymphocytes has been shown for many pathological conditions, including PerC inflammation [23], we studied if this molecule plays also a role in the orchestration of CD4^+^ T cells migration under steady state conditions. Our study using blocking antibodies against CXCL9, CXCL10 and CXCL11 (i.e., the known ligands of CXCR3) suggested that this molecule is not responsible for the preferential migration of cells to peritoneal cavity.

Previous studies have shown that the environment of the PerC results in changes in the gene expression profiles and migratory behavior of B cells [10], [11]. It has been also shown that the environment of other peripheral territories influences the expression profiles of T cells or DC. In our study we showed that the PerC environment changes the migratory properties of CD4^+^ cells and that after exposure to its milieu cells preferentially migrate back to this compartment. We also demonstrated that cells which were exposed to this environment up-regulate CXCR3 on their surface. However, the changes refer only to CXCR3^+^ cells and cells which do not express this molecule do not start to express it after exposure to the milieu of the PerC.

Since CXCR3 is considered as a Th1 marker, we assessed if the PerC is dominated by IFNγ producing cells. Indeed, around 40% of the CD4^+^ cells present in the PerC produce this cytokine. The percentage of CD4^+^ cells expressing IL-4 and IL-17 was also higher in this compartment. However, the differences in comparison to other organs were not as dominant as for the PerC. It is unlikely that the observed differences are due to differences in the proportion of memory cells in different organs, since our analysis of cytokine production by memory and naïve cells from Sp or PerC showed that memory cells from the PerC in general produce more cytokines. Thus, it seems that the PerC environment increases *per se* the level of expression of different cytokines, as suggested by the adoptive transfer experiments. An increase in IFNγ production is usually observed in CXCR3^+^ cells, whereas IL-4 production is mainly enhanced in the CXCR3^−^ subset. This suggests that the PerC is not changing the Th lineage, but rather promoting an overall increase in cytokine production levels. This interpretation of the data is fitting with the observation that the environment of the PerC increases the level of expression of CXCR3, but it does not induce its expression on cells which are negative for this chemokine receptor. However, the complete validation of this hypothesis is currently impossible, due to the lack of perfect markers for different Th lineages. Nevertheless, the obtained results led us to the conclusion that the observed domination of the PerC by Th1 cells is not due to the changes in Th lineage of cells after migration to this compartment, but rather due to a preferential migration of these cells to this niche. These data point to a new mechanisms for the environmental regulation of cytokine production, namely, as a result of differences in the migration of different Th cell subsets.

The data presented in this work bring also new light on the way in which we are defining the Th-lineage of CD4^+^ T cells. Currently cells are assigned to different Th-lineage due to the expression of signature cytokines. However, other factors such as the expression of transcription factors responsible for Th-differentiation are also taken into account. Our results show that definition according to cytokine production is problematic, since T cells can change their cytokine expression profiles as a result of changes in their anatomical localization. The PerC environment increases the expression of IFNγ mostly on CXCR3^+^ cells, which are considered as a Th1 cells, whereas up-regulation of IL-4 mainly occurs in the CXCR3^−^ compartment, which is considered as a Th2 lineage. These changes do not affect naïve cells, thereby suggesting that there is a commitment to produce particular cytokines by activated cells. Nevertheless, this commitment does not coerce cells to produce this cytokine, but rather confers them this potential.
